# Supplementary material for: SB365, Pulsatilla Saponin D Induces Caspase-Independent Cell Death and Augments the Anticancer Effect of Temozolomide in Glioblastoma Multiforme Cells
Source: Molecules. 2019 Sep 5;24(18):3230. doi: 10.3390/molecules24183230 (PMC6766801; doi:10.3390/molecules24183230)
Supplement: Supplementary file 1 [file molecules-24-03230-s001.pdf]

SB365, *Pulsatilla* Saponin D Induces Caspase-Independent Cell Death and Augments the Anticancer Effect of Temozolomide in Glioblastoma Multiforme Cells

Jun-Man Hong [1], Jin-Hee Kim [2], Hyemin Kim [3], Wang Jae Lee [1] and Young-il Hwang [1,*]

[1] Department of Anatomy and Cell Biology, Seoul National University College of Medicine, Seoul 03080, Republic of Korea
[2] Department of Biomedical Laboratory Science, Cheongju University, Cheongju 28503, Republic of Korea
[3] Research Institute for Future Medicine, Samsung Medical Center, Seoul 06351, Republic of Korea
* Correspondence: hyi830@snu.ac.kr; Tel.: 822-740-8209; FAX.: 822-745-9528

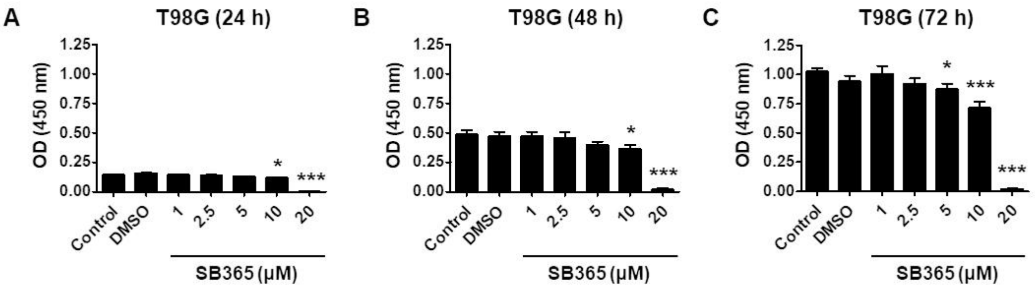

Figure S1. In vitro cytotoxic effect of SB365 on TMZ-resistant T98G cells.

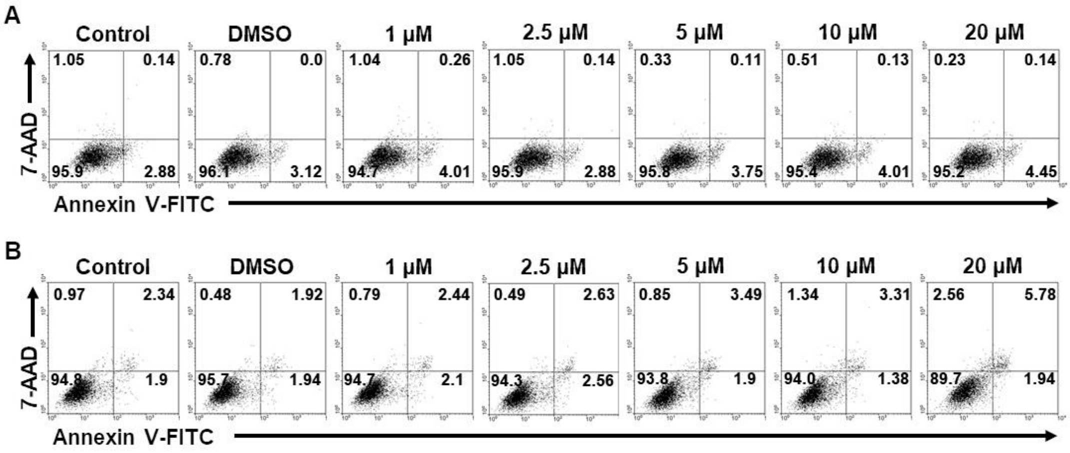

Figure S2. Cell death in U87-MG cells treated with SB365.

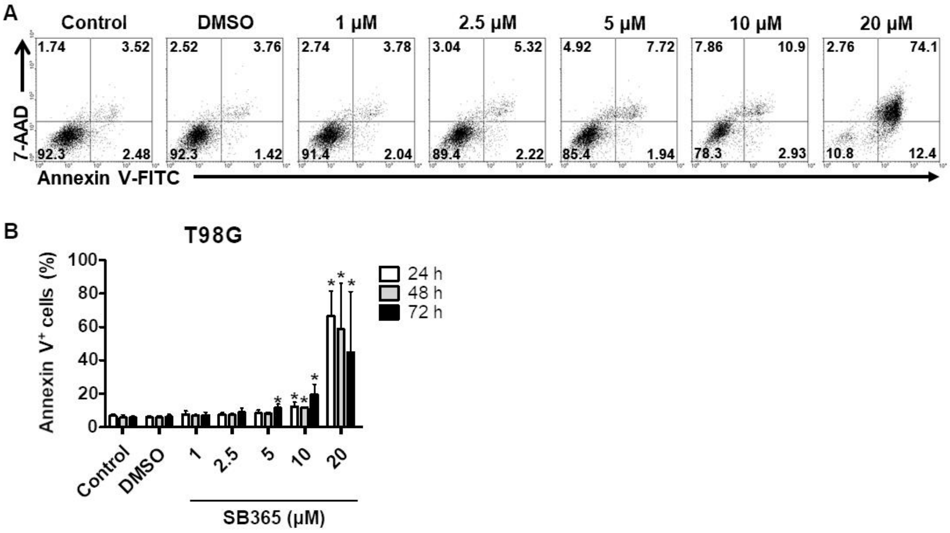

Figure S3. Cell death in T98G cells treated with SB365.

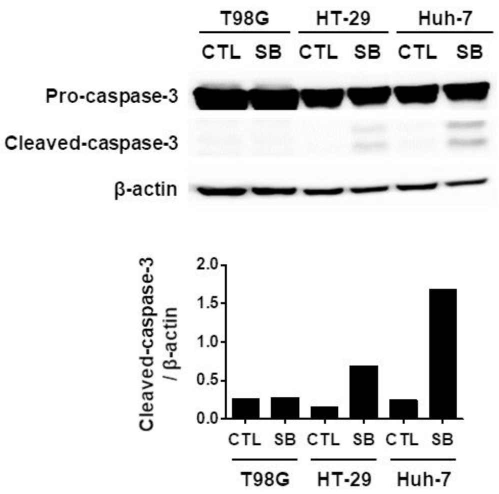

Figure S4. Effect of SB365 on the cleavage of caspase-3 in T98G cells.

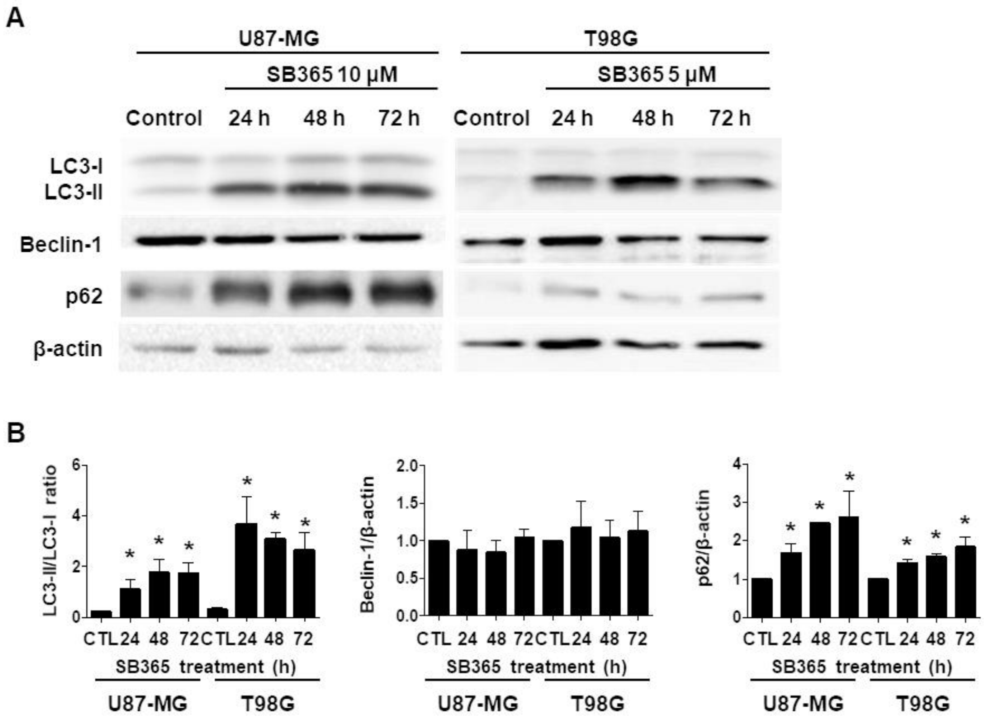

Figure S5. Effect of SB365 on the expression of autophagy-related proteins in GBM cells.

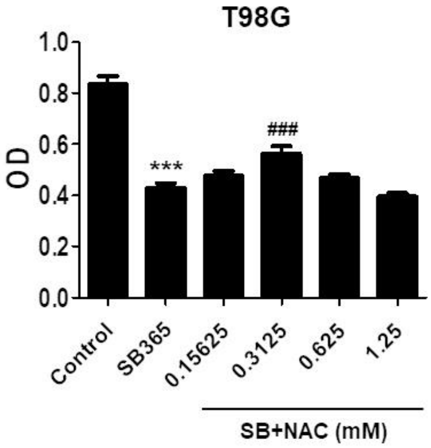

Figure S6. Recovery of SB365-induced cell death by antioxidant, NAC, in T98G cells.

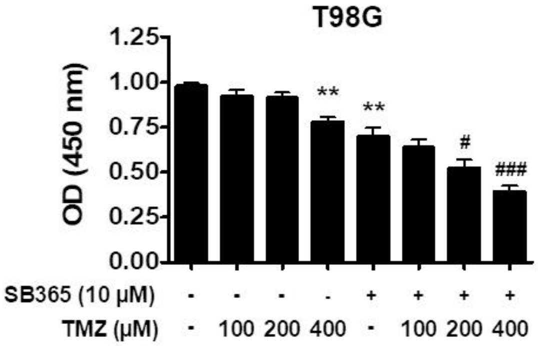

Figure S7. Cytotoxic effect of SB365 and TMZ on T98G cells.

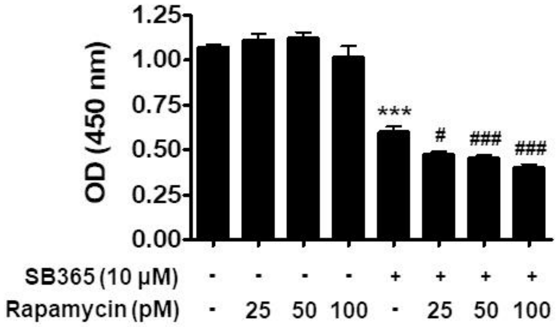

Figure S8. Augmentation of SB365 cytotoxicity by rapamycin pre-treatment.

Table S1. Cell proliferation in U87-MG cells treated with SB365 and/or TMZ.

**Viability reduction ratio in U87-MG cells (%)**		
	**TMZ only**	with SB365
Control	0 (±13)	28.7 (±12.2)
6.25 μM	5.9 (±17.7)	46.3 (±10)
12.5 μM	9.8 (±14.2)	48.5 (±10.1)
25 μM	36.8 (±8.3)	56.5 (±5.7)
50 μM	45.9 (±7.5)	63.6 (±9.9)
